# Age dependency of risk factors for cognitive decline

**DOI:** 10.1186/s12877-018-0876-2

**Published:** 2018-08-20

**Authors:** N. Legdeur, M. W. Heymans, H. C. Comijs, M. Huisman, A. B. Maier, P. J. Visser

**Affiliations:** 1 Alzheimer Center Amsterdam, Department of Neurology, Amsterdam Neuroscience, Vrije Universiteit Amsterdam, Amsterdam UMC, PO Box 7057, 1007 MB Amsterdam, the Netherlands; 2 Department of Epidemiology and Biostatistics, Amsterdam Public Health Research Institute, Vrije Universiteit Amsterdam, Amsterdam, the Netherlands; 3 GGZ inGeest / Department of Psychiatry, Amsterdam Public Health Research Institute, Vrije Universiteit Amsterdam, Amsterdam, the Netherlands; 4 Department of Sociology, Vrije Universiteit Amsterdam, Amsterdam, the Netherlands; 5 Department of Medicine and Aged Care, @AgeMelbourne, Royal Melbourne Hospital, University of Melbourne, Melbourne, Australia; 6 Department of Human Movement Sciences, @AgeAmsterdam, Faculty of Behavioural and Movement Sciences, Vrije Universiteit Amsterdam, Amsterdam, the Netherlands; 7 Department of Psychiatry & Neuropsychology, School for Mental Health and Neuroscience, Maastricht University, Maastricht, the Netherlands

**Keywords:** Cognitive decline, Risk factors, Aging, Oldest-old

## Abstract

**Background:**

Risk factors for cognitive decline might depend on chronological age. The aim of the study was to explore the age dependency of risk factors for cognitive decline in cognitively healthy subjects aged 55–85 years at baseline.

**Methods:**

We included 2527 cognitively healthy subjects from the Longitudinal Aging Study Amsterdam (LASA). Median follow-up was 9.1 (IQR: 3.2–19.0) years. The association of genetic and cardiovascular risk factors, depressive symptoms, inflammation markers and lifestyle risk factors with decline in MMSE and memory function was tested using spline regression analyses.

**Results:**

Subjects were on average 70.1 (SD 8.8) years old at baseline. Based on a spline regression model, we divided our sample in three age groups: ≤70 years (young-old), > 70–80 years (old) and > 80 years (oldest-old). The association of LDL cholesterol, homocysteine, hypertension, history of stroke, depressive symptoms, interleukin-6, a1-antichymotrypsin, alcohol use and smoking with cognitive decline significantly differed between the age groups. In general, the presence of these risk factors was associated with less cognitive decline in the oldest-old group compared to the young-old and old group.

**Conclusions:**

The negative effect of various risk factors on cognitive decline decreases with higher age. A combination of epidemiological factors, such as the selection towards healthier subjects during follow-up, but also risk factor specific features, for example ensuring the cerebral blood flow in case of hypertension, explain this diminished association at higher age. It is important to take these age differences into account when applying preventive strategies to avert cognitive decline.

**Electronic supplementary material:**

The online version of this article (10.1186/s12877-018-0876-2) contains supplementary material, which is available to authorized users.

## Background

Dementia is a growing health problem with an expected number of 115 million cases worldwide in 2050 [1]. The prevalence of dementia increases steeply with age from a prevalence of 2.6% in subjects aged 65–69 years and a prevalence of 43.1% in subjects aged 90 years and older [1]. Insight in the risk factors for cognitive decline is essential in the search for preventive strategies for cognitive impairment and dementia. Former studies identified a range of potential risk factors including the APOE (apolipoprotein E) ε4 allele, cardiovascular risk factors, depressive symptoms, inflammation markers and lifestyle factors [2–8]. However, whether the effect of risk factors on cognitive decline in cognitively healthy subjects is dependent on age is not clear, as the majority of the previous studies did not discriminate between younger and older subjects and the number of subjects aged 80 years and older in these studies was generally low [8, 9]. Still, there is increasing evidence that the association of risk factors with cognitive decline becomes less strong at higher age and may even have a protective effect [10]. For example, the risk of the APOE ε4 allele on Alzheimer’s dementia (AD) decreases after the age of 70 years [11]*.* In addition, the association of cardiovascular risk factors with cognitive decline might decrease with increasing age [10, 12–14].

The aim of the present study was to explore whether the association of risk factors with cognitive decline in cognitively healthy subjects across the age range of 55 to 85 years was dependent on age. We hypothesized that predictive accuracy would change with age for APOE ε4 allele and cardiovascular risk factors based on previous studies and we performed exploratory analyses to test whether age effects were also present for other established risk factors including depressive symptoms, inflammation markers, alcohol use, smoking and physical activity.

## Methods

### Study sample

Data were derived from the on-going Longitudinal Aging Study Amsterdam (LASA) [15]. This is a longitudinal, population-based study in the Netherlands focusing on trajectories of physical, psychological, social and cognitive functioning in subjects aged 55 years and older. In 1992–1993 a random sample of men and women aged 55–85 years, stratified for age and sex, from three geographic areas of the Netherlands (Amsterdam, Zwolle and Oss) was included. Follow-up measurements were conducted about every 3 years. Data collection included a main and medical interview conducted in the homes of the subjects. The main interview was done by trained and supervised interviewers and the medical interview was performed by trained nurses. All subjects gave informed consent and the study was approved by the Ethical Review Board of the VU University Medical Center (VUmc), Amsterdam, the Netherlands and conducted according to the principles of the Helsinki declaration.

At the start of the study in 1992–1993, 3107 subjects were enrolled. To select cognitively healthy subjects at baseline, subjects with an age and education corrected MMSE lower than 27 points were excluded (this cut-off is based on the lowest 10th percentile of the MMSE in the Maastricht Aging Study (MAAS) [16], leaving 2527 subjects at baseline. In 1995–1996, 2545 subjects were re-examined. See for further details about the following cycles of this LASA cohort and for the sample size per risk factor Additional file 1: Table S1 and S2.

### Measurements

#### Biomaterial

The ApoE phenotyping was done either in 1992–1993 or 1995–1996 at the Immunochemisch Laboratorium of the VUmc. The blood samples were frozen at -80 °C until determination in 1997–1998. The method used is described by Havekes et al. (1987) and consisted of isoelectric focusing of delipidated serum samples, followed by immunoblotting [17]. In the analyses, we used the presence of an ApoE ε4 isoform (phenotypes ε2/4, ε3/4, ε4/4) as a dichotomous variable [17]. The ApoE ε4 isoform was used as proxy for the presence of an APOE ε4 allele.

Cholesterol levels (total cholesterol, High-Density Lipoprotein (HDL) cholesterol and Low-Density Lipoprotein (LDL) cholesterol) and homocysteine (in combination with vitamin B12) were determined in morning blood samples collected in 1995–1996 (second LASA cycle). Subjects were allowed to eat toast and drink tea, but no dairy products. The EDTA plasma samples were stored at -80 °C and analyzed by the Department of Clinical Chemistry of the VUmc in 2001/2002 (homocysteine) and 2005 (cholesterol). For determination of total cholesterol and HDL cholesterol enzymatic colorimetric tests were used. LDL cholesterol was calculated as total cholesterol minus HDL-cholesterol minus VLDL-cholesterol; VLDL-cholesterol was calculated as total triglyceride concentration expressed in mmol/L multiplied by 0.456 [18]. This method is less reliable when the triglyceride level is ≥5.0 mmol/L. Therefore, this analysis was only done for triglyceride levels of < 5.0 mmol/L. Total homocysteine was determined with the Abbott IMx analyser which uses fluorescence polarization immunoassay (FPIA) technology. Serum levels of vitamin B12 were determined at the Endocrine Laboratory of the VUmc with a competitive immunoassay luminescence on the automated ACS 180 System (Bayer Diagnostics, Mijdrecht, The Netherlands).

For determination of the inflammation markers (interleukin-6 (IL-6), C-reactive protein (CRP) and a1-antichymotrypsin (ACT)) serum collected in 1992–1993 (only in Amsterdam and Zwolle) was stored at -80 °C until determination in 2002–2004. Sensitive regular immunoassays (ELISA) were used at Sanquin Research (Amsterdam) to determine IL-6, CRP and ACT. CRP was expressed in ug/ml, IL-6 in pg/ml and ACT in % of normal plasma. The normal human plasma pool (% NHP) used as a standard for ACT contained ~ 300 mg ACT per L. For part of the subjects, CRP levels were determined directly after blood sampling.

Both the cholesterol as the inflammation markers, were added to the analyses as continuous variables.

#### Comorbidity

Hypertension was defined as a blood pressure > 140/90 mmHg measured at the upper arm or the use of antihypertensive medication collected in the first follow-up measurement in 1995–1996 (at baseline blood pressure was measured only at the finger). Post-hoc we also analyzed the association of a measured high blood pressure and the use of antihypertensive medication with cognitive decline separately.

The presence of a history of myocardial infarction (MI), DM or stroke was assessed by self-report. The assessment of comorbidity by self-report was found to be comparable with the medical information reported by the general practitioner [19].

Depressive symptoms were assessed using the Center for Epidemiologic Studies Depression scale (CES-D) [20]. The CES-D is a self-report scale containing 20 items describing depressive symptoms. The maximum score is 60 with higher scores indicating more depressive symptoms. In the analyses, the CES-D was used as a continuous variable.

#### Lifestyle

The number of alcohol consumptions was categorized into three categories: 0 alcoholic drinks per day (‘none’ group), 1–2 alcoholic drinks for men and 1 alcoholic drink for women per day (‘minimal’ group) or > 2 alcohol drinks for men and > 1 alcohol drink for women per day (‘moderate’ group) [8]. Smoking was dichotomized in ‘yes (or stopped within one year)’ or ‘no’.

For the assessment of physical activity, the LASA Physical Activity Questionnaire (LAPAQ) was used addressing walking outdoors, bicycling, light household, heavy household, and two sports activities [21]. The subjects are asked how often and how long they carried out these activities in the past 2 weeks. In the analyses, total physical activity in minutes per day was used as a continuous variable.

#### Cognitive outcome measures

Two different neuropsychological tests were used as outcome measures: The Mini-Mental State Examination (MMSE) and 15 Words Test (15WT). The MMSE is the most used screening instrument for global cognitive dysfunction [22]. The score ranges from 0 to 30 points, with higher scores indicating better cognitive functioning. The 15WT is the Dutch version of the Auditory Verbal Learning Test [23]. Fifteen words have to be learned over five trials. In LASA the 15WT is restricted to three trials due to a limitation in time. In this study we used the maximum immediate recall score and delayed recall score, both ranging from 0 to 15 words. The delayed recall was assessed after 20 minutes of distraction.

### Statistical analyses

#### Spline regression analyses

Former studies, have shown that the association between age and cognition is nonlinear [24]. Linear regression techniques are therefore not sufficient enough to estimate this association and spline regression analyses are indicated to fit the nonlinear longitudinal associations between age and the cognitive outcome measures more precisely (Additional file 2: Spline regression analyses) [25]. To achieve the best fit of the data with a spline regression model, either linear or cubic splines can be used. Based on the likelihood-ratio (LR) test we determined which of these two types of splines showed a better fit with our data. We examined the positions where the splines join smoothly together, referred to as knots in spline regression analyses. We identified the optimal position of the knots by testing both a model with one and two knots and moving those 5 years up and down. The ages that corresponded to the position of the knots were used to separate our sample into different age groups, to facilitate interpretation of results. Lastly, for all the different risk factors and outcome measures we compared our final model with a linear regression model without splines to test whether the model with splines showed a better fit (based on the LR test).

#### Differences between age groups at baseline

Statistically significant differences in baseline characteristics between the age groups were determined by using ANOVA for continuous variables, chi-square for categorical variables and Kruskal-Wallis test for skewed variables. Mixed model analysis was used to determine the difference in cognitive test score change per year in the age groups.

#### Association of risk factors with cognitive outcome measures

We performed three different analyses to determine the association of the risk factors (measured at baseline or, for hypertension and some biomaterial measurements, in the second cycle) with the cognitive outcome measures. In all these analyses, splines (determined as described in 2.3.1) were added to the model to estimate the association between age and the cognitive outcome measure. First, we determined the association of the risk factors with the three cognitive outcome measures in the total sample by using a linear mixed model (including a random intercept and fixed slopes). Secondly, we added the interaction of the risk factors with the splines to the analyses to assess the age dependency of the risk factors. Because the splines represent different age groups, a significant interaction means that the association of that risk factor with the cognitive outcome measure is different between age groups. If this interaction was statistically significant for a categorized risk factor, we visualized the association in a figure. Lastly, we determined the association coefficient per age group of the risk factors with the cognitive outcome measures. This last step helps us to interpret the results we found with the interaction analyses (we also performed these analyses for the risk factors that did not show a significant interaction). All the analyses were adjusted for sex and education (in years).

#### Selection during follow-up

To determine whether there was a selection towards healthier subjects during follow-up, we determined the *baseline* values of the risk factors and cognitive outcome measures of the subjects that were present in the sample during each LASA cycle. Decreasing baseline values during follow-up would be indicative of selection towards healthier subjects.

#### Statistical software

The spline regression analyses were performed with the statistical software R version 3.2.5 (http://www.r-project.org). The statistical significance of the association of the risk factor with cognitive decline per age group was determined with Stata version 15. The differences in baseline characteristics between the three age groups were analyzed with SPSS Statistics version 22. The level of significance was set to *p* = 0.05.

## Results

We included 2527 subjects (51.2% women) who were on average 70.1 (SD: 8.8, range: 54.8–85.6) years at baseline and had 9.1 (SD: 3.4) years of education (Table 1). Median follow-up was 9.1 (IQR: 3.2–19.0) years.

**Table 1:** Baseline characteristics of subjects in the total sample

Characteristic	Total sample	≤70 years	> 70–80 years	> 80 years	*P* *-value* ^b^
Sample size^a^	2527	1292	794	441	
Age, y	70.1 (8.8)	62.6 (4.2)	75.5 (2.9)	82.6 (1.5)	
Female, %	51.2	52.3	50.0	50.3	*0.48*
Education, y	9.1 (3.4)	9.5 (3.3)	8.7 (3.2)	8.6 (3.7)	*< 0.01*
Follow-up, y (median, IQR)	9.1 (3.2–19.0)	13.3 (8.9–19.2)	6.2 (3.0–13.0)	4.9 (3.3–8.9)	*< 0.01*
MMSE, points (median, IQR)	28 (27–29)	29 (26–30)	28 (24–30)	27 (24–30)	*< 0.01*
Change in MMSE per year (SE)	−0.11 (0.00)	− 0.06 (0.00)	− 0.18 (0.01)	− 0.25 (0.02)	*< 0.01*
15WT immediate recall, words	8.1 (2.5)	8.9 (2.3)	7.6 (2.4)	6.3 (2.1)	*< 0.01*
Change in 15WT immediate recall per year (SE)	−0.11 (0.00)	−0.07 (0.00)	− 0.11 (0.01)	−0.07 (0.02)	*< 0.01*
15WT delayed recall, words	5.3 (2.7)	6.2 (2.6)	4.7 (2.5)	3.5 (2.2)	*< 0.01*
Change in 15WT delayed recall per year (SE)	−0.11 (0.00)	−0.05 (0.01)	− 0.10 (0.01)	−0.06 (0.02)	*< 0.01*
APOE ε4, %^c^	26.3	27.8	25.2	23.6	*0.29*
Total cholesterol, mmol/L	5.7 (1.0)	5.9 (1.0)	5.6 (1.0)	5.3 (1.1)	*< 0.01*
LDL cholesterol, mmol/L	3.7 (1.0)	3.8 (0.9)	3.6 (0.9)	3.4 (1.1)	*< 0.01*
HDL cholesterol, mmol/L	1.3 (0.4)	1.3 (0.4)	1.4 (0.4)	1.3 (0.4)	*0.48*
Homocysteine, mmol/L	14.5 (6.1)	13.5 (5.2)	14.7 (5.0)	17.2 (9.2)	*< 0.01*
Vitamin B12, pMol/L (median, IR)	266 (212–333)	268 (219–335)	264 (213–331)	249 (196–333)	*0.12*
Hypertension, %	76.7	72.9	82.2	73.7	*< 0.01*
Myocardial infarction, %	8.8	6.4	11.1	11.7	*< 0.01*
Diabetes mellitus, %	7.0	4.3	8.6	12.3	*< 0.01*
Stroke, %	4.4	1.6	6.7	8.7	*< 0.01*
CES-D total score (median, IQR)	5 (2–11)	5 (2–9)	6 (3–11)	7 (3–12)	*< 0.01*
IL-6, pg/ml (median, IQR)	1.4 (0.6–2.5)	1.3 (0.6–2.4)	1.6 (0.7–2.7)	1.8 (1.0–3.1)	*< 0.01*
CRP, ug/ml (median, IQR)	2.2 (1.0–4.7)	2.0 (0.9–3.9)	2.5 (1.3–5.6)	2.8 (1.4–5.7)	*< 0.01*
ACT, % of NHP	173.6 (57.6)	169.6 (53.0)	179.3 (66.1)	177.2 (54.1)	*0.01*
Alcohol consumption, %					
None	20.1	15.6	25.3	24.7	*< 0.01*
Minimal^d^	20.6	17.8	23.6	23.6	
Moderate^e^	59.4	66.6	51.1	51.7	
Smokers, %	24.6	30.4	23.1	19.8	*< 0.01*
Total physical activity, min per day	169.2 (114.2)	188.4 (121.3)	160.0 (103.7)	126.8 (95.3)	*< 0.01*

*15WT* 15 Words Test, *ACT* a1-antichymotrypsin, *APOE* apolipoprotein E, *CES-D* Center for Epidemiologic Studies Depression scale, *CRP* C-reactive protein, *HDL* High-Density Lipoprotein, *IL-6* interleukin-6, *IQR* interquartile range, *LASA* Longitudinal Aging Study Amsterdam, *LDL* Low-Density Lipoprotein, *MMSE* Mini-Mental State Examination, *NHP* normal human plasma, *SE* standard error

^a^Sample size varies per characteristic (Additional file 1: Table S2). ^b^Differences between the three age groups tested with ANOVA for continuous variables, chi-square for categorical variables, Kruskal-Wallis test for non-parametric variables and mixed model analysis for the change in cognitive test scores per year. ^c^Percentage of subjects with an apolipoprotein E ε4 isoform as proxy for an APOE ε4 allele. ^d^Women:1 drink/day, men: 1–2 drinks/day. ^e^Women: > 1 drink/day, men: > 2 drinks/day. Values are means (SD) unless stated otherwise

### Determination of the best-fitted spline regression model

**Fig. 1:**
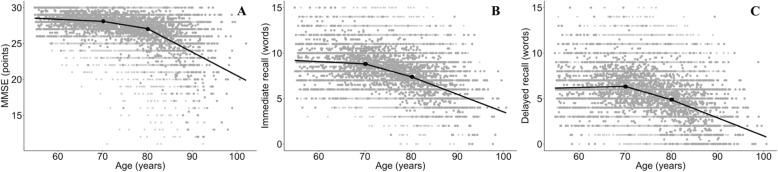
Association between age and Mini-Mental State Examination (MMSE, figure **a**) and 15 Words Test (15WT, figure **b** and **c**). The grey dots represent all the longitudinal data points. The lines represent the splines with the two knots (black dots) at the ages 70 and 80 years

For the longitudinal associations between the cognitive outcome measures and age in the total group, a linear spline regression model showed a better fit then a cubic spline regression model. A model with two knots placed at the ages 70 and 80 years showed the best fit for the three outcome measures, dividing the sample in three age groups: ≤70 years (young-old subjects), > 70–80 years (old subjects) and > 80 years (oldest-old subjects) (Fig. 1). The spline regression model showed a better fit then a linear regression model without splines for all the different associations.

### Differences between age groups at baseline

Most characteristics differed between age groups (Table 1). Years of education, follow-up time, scores on the cognitive tests, total and LDL cholesterol levels, alcohol use, smoking and physical activity all decreased with age. The level of homocysteine and inflammation markers and the presence of cardiovascular comorbidities and depressive symptoms increased with age.

### Association of risk factors with cognitive decline in the total sample

In the total sample, the presence of high homocysteine levels, history of stroke and depressive symptoms were associated with more decline in MMSE and the 15WT (Table 2). Alcohol use was associated with less decline in MMSE and the 15WT. The presence of APOE ε4 was associated with more decline in MMSE and a history of DM with more decline in the 15WT. Cholesterol levels, hypertension, history of MI, inflammation markers, smoking and physical activity were not associated with cognitive decline.

**Table 2:** The association of risk factors with cognitive decline in the total sample

Risk factor	MMSE			15WT immediate recall			15WT delayed recall		
	B	SE	*P-value*	B	SE	*P-value*	B	SE	*P-value*
APOE ε4	−0.25	0.24	*< 0.01*	−0.07	1.27	*0.49*	−0.09	1.44	*0.42*
Total cholesterol	0.05	0.17	*0.34*	0.09	1.22	*0.11*	0.08	1.39	*0.21*
LDL cholesterol	0.05	0.17	*0.41*	0.08	1.24	*0.20*	0.08	1.41	*0.25*
HDL cholesterol	0.16	0.16	*0.20*	0.23	1.21	*0.08*	0.07	1.42	*0.67*
Homocysteine	−0.05	0.12	*< 0.01*	−0.05	1.16	*< 0.01*	− 0.06	1.33	*< 0.01*
Hypertension	0.13	0.28	*0.26*	−0.02	1.26	*0.88*	−0.03	1.43	*0.85*
DM	−0.16	0.29	*0.25*	−0.42	1.28	*0.01*	−0.42	1.45	*0.04*
MI	0.05	0.29	*0.69*	0.08	1.28	*0.58*	0.22	1.48	*0.21*
Stroke	−0.40	0.28	*0.02*	−0.55	1.27	*0.01*	−0.58	1.44	*0.02*
Depressive symptoms	−0.01	0.33	*< 0.01*	−0.02	1.33	*< 0.01*	−0.02	1.50	*< 0.01*
CRP	0.00	0.27	*0.57*	−0.01	1.27	*0.15*	−0.01	1.45	*0.47*
IL-6	0.01	0.26	*0.46*	0.01	1.27	*0.72*	0.02	1.44	*0.42*
ACT	0.00	0.27	*0.68*	0.00	1.27	*0.74*	0.00	1.45	*0.87*
Alcohol^a^: minimal^b^	0.27	0.10	*< 0.01*	0.35	0.12	*< 0.01*	0.35	0.14	*0.01*
Alcohol^a^: moderate^c^	0.24	0.08	*< 0.01*	0.41	0.10	*< 0.01*	0.42	0.12	*< 0.01*
Smoking	−0.09	0.25	*0.26*	−0.16	1.27	*0.09*	−0.03	1.46	*0.77*
Physical activity	0.00	0.30	*0.78*	0.00	1.28	*0.59*	0.00	1.45	*0.24*

B’s are determined by linear mixed models in combination with splines and adjusted for sex and education

*15WT* 15 Words Test, *ACT* a1-antichymotrypsin, *APOE* apolipoprotein E, *CRP* C-reactive protein, *DM* Diabetes mellitus, *HDL* High-Density Lipoprotein, *IL-6* interleukin-6, *LDL* Low-Density Lipoprotein, *MI* Myocardial infarction, *MMSE* Mini-Mental State Examination

^a^No alcohol use is reference group. ^b^Women:1 drink/day, men: 1–2 drinks/day. ^c^Women: > 1 drink/day, men: > 2 drinks/day

### Age dependency of risk factors

**Fig. 2:**
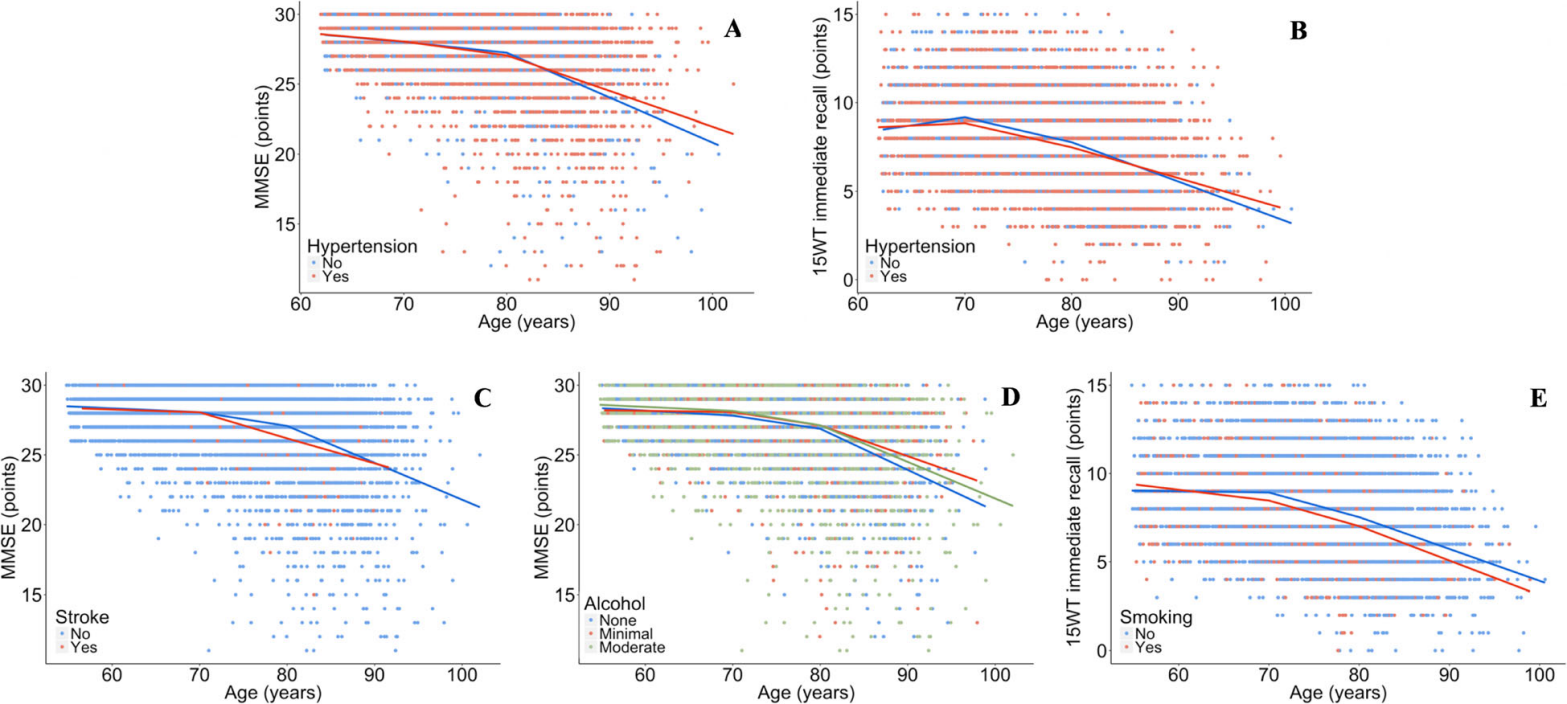
Association of risk factors with Mini-Mental State Examination (MMSE, figure **a**, **c** and **d**) or 15 Words Test (15WT) immediate recall (figure **b** and **e**). Shown are the categorized risk factors (hypertension, stroke, alcohol use (‘minimal’: 1 drink/day for women and 1–2 drinks/day for men, ‘moderate’: > 1 drink/day for women and > 2 drinks/day for men) and smoking) which have a significant age-dependent association with the outcome measure (MMSE or 15WT immediate recall). B’s can be found in Table 3

The association of LDL cholesterol, homocysteine, hypertension, history of stroke, depressive symptoms, IL-6, ACT, alcohol use and smoking with cognitive decline differed between the age groups (Table 3 and Fig. 2). The presence of APOE ε4, total and HDL cholesterol level, a history of DM or MI, CRP level and physical activity did not show an age effect. In general, the regression coefficient changed from a negative association in the young-old and old subjects to a positive association in the oldest-old subjects. This means that on top of the decline in MMSE and 15WT as visualized in Fig. 1, the presence of these risk factors was associated with more decline in MMSE or 15WT in the young-old and old subjects and less decline in MMSE or 15WT in the oldest-old subjects. If we determined the association of the age-dependent risk factors with cognitive decline per age group, we found that hypertension, high IL-6 levels, and alcohol use were significantly associated with less cognitive decline in the oldest-old subjects (Table 3 and Additional file 1: Tables S5–S6). Smoking was significantly associated with more memory decline in the young-old subjects and high LDL cholesterol with more MMSE decline in the young-old subjects (Table 3 and Additional file 1: Table S6).

**Table 3:** The association of risk factors with cognitive decline per age group

Risk factor	MMSE			15WT immediate recall			15WT delayed recall		
	≤70	> 70–80	> 80	≤70	> 70–80	> 80	≤70	> 70–80	> 80
APOE ε4	0.72	**−4.24**	**−10.17**	−0.65	**−3.85**	**−5.32**	0.18	**−4.15**	**−8.52**
Total cholesterol	−3.37	−0.10	1.26	−1.08	− 1.09	1.53	0.97	−1.53	1.27
LDL cholesterol	−5.40*	0.66	1.83*	−0.18	−1.92*	1.96*	0.68	**−2.62**	1.41
HDL cholesterol	7.35	−2.17	−1.07	− 1.84	2.64	0.50	6.21	1.45	2.97
Homocysteine	0.39	−0.40	**−0.92**	− 0.39	− 0.21	0.13	− 0.76*	−0.08	0.13*
Hypertension	0.04	−2.14*	6.52*	−5.65*	−0.22	5.06*	−4.04	3.54	0.43
DM	−0.95	−0.46	−9.32	− 0.11	−3.49	−0.29	−1.68	−5.44	4.90
MI	2.42	−1.59	2.23	0.41	1.93	5.64	4.90	0.62	**10.14**
Stroke	1.25	−9.16*	9.16*	2.60	−0.34	6.70	0.79	−1.58	−4.30
Depressive symptoms	−0.07	0.02	−0.06	−0.21	0.00	0.07	−0.11*	−0.23	0.21*
CRP	−0.18	0.18	0.19	−0.21	0.03	0.19	−0.19	−0.11	0.16
IL-6	0.02*	−0.10*	1.31*	0.27	−0.11	0.64	0.31	−0.03	0.88
ACT	0.00	0.01	**0.06**	−0.01	− 0.01	0.03	− 0.01*	−0.04	0.03*
Alcohol^a^: minimal^b^	2.82*	−0.26	7.93*	3.19	−3.09	−0.31	5.91	−3.03	0.25
Alcohol^a^: moderate^c^	0.78	−0.95	3.48	2.85	−3.51	1.42	3.19	−0.47	−0.69
Smoking	−0.30	−1.05	−5.15	−5.84*	0.53*	−1.13	**−4.52**	−2.88	−0.61
Physical activity	0.00	0.00	**0.02**	−0.01	0.01	0.00	0.01	0.01	0.00

Shown are beta’s (multiplied by 100) of the associations of a risk factor with cognitive decline within each age group. They show the extra cognitive decline (next to the overall cognitive decline as visualized in Fig. 1) per age group in the presence of a risk factor. A negative beta indicates that a unit increase in the risk factor is associated with more cognitive decline. Bold beta’s indicate a significant (*p* < 0.05) association with cognitive decline in that age group (in Additional file 1: Tables S5–S7 we present the standard errors and *p*-values corresponding to the beta’s in this table per age group)

*15WT* 15 Words Test, *ACT* a1-antichymotrypsin, *IL-6* interleukin-6, *LDL* Low-Density Lipoprotein, *MMSE* Mini-Mental State Examination

*Association of risk factor with MMSE or 15WT decline is significantly different between these two age groups. In case of three *: difference is significant between ≤70 and > 80 years old group and between > 70–80 and > 80 years old group. Beta’s are determined by linear mixed models in combination with splines and adjusted for sex and education

^a^No alcohol use is reference group. ^b^Women:1 drink/day, men: 1–2 drinks/day. ^c^Women: > 1 drink/day, men: > 2 drink/day

Post-hoc analyses with hypertension defined by the measured high blood pressure only or the use of antihypertensive medication only, yielded similar results (Additional file 1: Table S3).

### Selection during follow-up

Subjects who were retained in the later LASA cycles had a lower age, higher level of education, higher scores on the cognitive outcome measures, higher cholesterol levels, less comorbidities, lower levels of inflammation markers, higher alcohol use, lower levels of smoking and had a higher level of physical activity at baseline compared to subjects who dropped out during follow-up (Additional file 1: Table S4).

## Discussion

This study showed that the association of LDL cholesterol, homocysteine, hypertension, history of stroke, depressive symptoms, IL-6, ACT, alcohol use and smoking with cognitive decline was age-dependent. In general, these risk factors were associated with more cognitive decline in the young-old and old subjects and less cognitive decline in the oldest-old subjects. APOE ε4 genotype and DM were negatively associated with cognitive decline regardless of age.

Before we discuss these findings in more detail, it should be noted that the baseline age of the subjects with follow-up was lower than that of the subjects who were lost to follow up. The subjects with follow-up also showed a better overall health with less comorbidity. The selection towards younger and healthier subjects at follow-up may explain why the negative impact of the risk factors was strongest in younger subjects. However, it does not explain why these risk factors became protective at higher age. We reduced the potential selection bias by combining baseline data and follow-up data across the age span in the spline regression model. In this way follow-up data of the selected younger, healthier subjects (Additional file 1: Table S4) were combined with baseline data of the older, less healthy subjects (Table 1).

In addition, most subjects dropped out of the study because of mortality [15]. A comparison of one-year mortality rates of LASA subjects with those in the general Dutch population showed that mortality in the LASA subjects was slightly higher than in the general population, but that this difference exceeded 1% only in women aged 80–85 years (unpublished data). Therefore, it may not necessarily affect the generalizability of our findings.

### APOE genotype

Our finding that the APOE ε4 genotype increases the risk for cognitive decline regardless of age is at odds with some earlier studies that reported a decrease of risk for dementia with age [11, 26] but is consistent with others [27]. Differences between studies may be explained by differences in selection of subjects (normal cognition or MCI) and outcome measure (progression to dementia or cognitive decline).

### Cardiovascular factors

Most cardiovascular factors were associated with less decline at higher age than at younger age, which is in line with previous studies [12, 28]. High cholesterol in late-life can be an indicator of a better nutritional status and a better overall health and therefore associated with less cognitive decline [28, 29]. Additionally, cholesterol synthesis is thought to decrease with aging, but only in AD patients and not in subjects with a normal cognition [30]. The association of low cholesterol with more cognitive decline in the oldest-old subjects might therefore be an expression of underlying AD pathology.

Hypertension may prevent cognitive decline at old age by ensuring the cerebral blood flow [31, 32]. On the other hand, low blood pressure can be a consequence of neurodegenerative disease and therefore an early sign of dementia onset, although this is an aspect which one can also expect in the young-old subjects [33, 34].

### Depressive symptoms

Earlier studies have shown that depressive symptoms are an important risk factor for cognitive decline and dementia, also among the oldest-old subjects [7, 35]. We replicated this finding for the total sample but also found that the association of depressive symptoms with memory decline was less in the oldest-old group than in the young-old group. This may be explained by the fact that older subjects score higher on the CES-D questionnaire for reasons other than depression, such as somatic morbidity [36]. In line with this explanation, earlier cross-domain latent growth models on LASA data demonstrated that delayed recall was associated with increasing levels of depressed affect, but not with depressive somatic symptoms [37].

### Inflammation markers

Inflammation has been described as an important mechanism underlying cognitive decline but most of these studies were performed in younger subjects [38]. We did not find an association of inflammation markers with cognitive decline in the total sample but noted differences between age groups showing that higher IL-6 and ACT levels were associated with less cognitive decline in subjects aged 80 years and older compared to younger subjects. Potentially, higher inflammation markers in older subjects are a sign of a better inflammatory response and therefore related to better overall health and cognitive functioning.

### Lifestyle factors

Minimal and moderate alcohol use were positively associated with decline in MMSE and memory functioning compared to no alcohol use, which is in line with earlier studies [3, 8]. We found that the positive association of alcohol use with cognition was strongest in subjects aged 80 years and older. In this age group, no alcohol use is frequently related to poor physical functioning. Therefore, the negative association of no alcohol use with cognitive decline is potentially an indirect effect [39].

In accordance with our findings, a meta-analysis in 2015 showed that the negative association of smoking with cognitive decline is decreasing with age [40]. Survival bias and the presence of competing risks are probably the most important phenomena to explain this finding [40–42].

In contrast to a meta-analysis of observational studies, we did not find an association between physical activity and cognitive decline [43]. However, a meta-analysis of intervention studies on the effect of aerobic exercise on cognitive decline in cognitively normal subjects did not find an effect [44]. We found that in the oldest age group less physical activity was associated with more cognitive decline, but because the over age interaction effect was not statistically significant, this finding should be interpreted cautiously.

### Cognitive outcome measures

We used different cognitive tests as outcome measure (MMSE, 15WT total and delayed recall) and in the total sample, most variables showed similar findings for the different outcome measures and the course of the tests was very similar with age (Table 2 and Fig. 1). However, we also found different results for the different outcomes, which may be explained by the fact that the tests measure different disease processes; memory decline is presumed to be an early marker of Alzheimer’s disease and decline in MMSE can be caused by a broader range of diseases [45].

### Strengths and limitations

This is the first study that analyzes the influence of age on the association of different types of risk factors with cognitive decline in one prospective cohort study. Earlier studies have indicated age differences but never studied the various risk factors in one cohort. Additionally, the use of a nonlinear analyze technique, is an important added value of this study to earlier literature. A limitation of this study is that we did not have data about the presence of cardiovascular risk factors, such as hypertension and high cholesterol, before age 55 years. Therefore, we could not discriminate between a high blood pressure and cholesterol emerging at high age or already present at younger ages. While we tested many risk factors with different outcomes at the same time, this also increased the risk of false-positive findings. However, we decided not to correct for multiple testing given the exploratory nature of the study and the increased risk of missing important findings when applying Bonferroni adjustments (type II errors) [46, 47]. For simplification purposes, we describe our results in relation to cognitive decline, although we only used the MMSE and 15WT to assess cognition. It therefore needs to be noted that these results cannot automatically be extrapolated to other forms of cognition. In addition, the 15WT is a test for verbal episodic memory and does not assess other types of memory such as visual and semantic memory.

### Scientific and clinical implications

With this study we showed that age needs to be taken into consideration when studying risk factors for cognitive decline. It is not only needed to add age as a confounder but especially as an effect modifier to analyses as it changes the relation between a risk factor and cognitive decline. From a clinical perspective, these results suggest that different preventive strategies might be effective in young-old versus oldest-old subjects. Antihypertensive and cholesterol lowering medication might not be appropriate for the oldest-old subjects who develop hypertension and hypercholesterolemia at a high age.

## Conclusions

The associations of LDL cholesterol, homocysteine, hypertension, history of stroke, depressive symptoms, IL-6, ACT, alcohol use and smoking with cognitive decline were different per age group. They were all less strongly associated with cognitive decline in the older subjects compared to younger subjects. Selection towards healthier subjects during follow-up need to be considered as possible explanation but also risk factor specific considerations, such as ensuring the cerebral blood flow in case of hypertension, need to be taken into account. These age differences are important when applying preventive strategies to avert cognitive decline.

## Additional files

Additional file 1: Additional tables about sample sizes, the association of hypertension with cognitive decline separately for use of antihypertensive medication and blood pressure, baseline characteristics per LASA cycle and the associations of the risk factors with the three cognitive tests in the three age groups. (DOCX 72 kb)

Additional file 2: Additional explanation about the spline regression analyses. (DOCX 375 kb)

## Abbreviations

15WT: 15 Words Test
ACT: A1-antichymotrypsin
AD: Alzheimer’s dementia
APOE: Apolipoprotein E
CES-D: Center for Epidemiologic Studies Depression scale
CRP: C-reactive protein
DM: Diabetes mellitus
HDL cholesterol: High-Density Lipoprotein cholesterol
IL-6: Interleukin-6
IQR: Interquartile range
LAPAQ: LASA Physical Activity Questionnaire
LASA: Longitudinal Aging Study Amsterdam
LDL cholesterol: Low-Density Lipoprotein cholesterol
LR: Likelihood-ratio
MAAS: Maastricht Aging Study
MI: Myocardial infarction
MMSE: Mini-Mental State Examination
NHP: Normal human plasma
SD: Standard deviation
SE: Standard error
VLDL cholesterol: Very-low-density lipoprotein cholesterol
VUmc: VU University Medical Center
