# Supplementary material for: Age dependency of risk factors for cognitive decline
Source: BMC Geriatr. 2018 Aug 20;18:187. doi: 10.1186/s12877-018-0876-2 (PMC6102935; doi:10.1186/s12877-018-0876-2)
Supplement: Supplementary file 1 — Additional tables about sample sizes, the association of hypertension with cognitive decline separately for use of antihypertensive medication and blood pressure, baseline characteristics per LASA cycle and the associations of the risk factors with the three cognitive tests in the three age groups. (DOCX 72 kb) [file 12877_2018_876_MOESM1_ESM.docx]

**Additional file 1: Tables**

**Table S1. Sample size per LASA cycle**

|  | LASA cycle 1992-1993 | LASA cycle  1995-1996 | LASA cycle 1998-1999 | LASA cycle  2001-2002 | LASA cycle  2005-2006 | LASA cycle  2008-2009 | LASA cycle  2011-2012 |
| --- | --- | --- | --- | --- | --- | --- | --- |
| Total | 2527 | 2148 | 1782 | 1482 | 1122 | 891 | 708 |
| MMSE | 2527 | 1959 | 1621 | 1297 | 945 | 762 | 571 |
| 15WT immediate recall | 2196 | 1237 | 1174 | 1146 | 868 | 672 | 500 |
| 15WT delayed recall | 2196 | 1236 | 1167 | 1143 | 857 | 653 | 490 |

*15WT* 15 Words Test, *LASA* Longitudinal Aging Study Amsterdam, *MMSE* Mini-Mental State Examination.

**Table S2. Sample size specified per age group, outcome and risk factor**

| Risk factor | ≤70 year olds | | >70-80 year olds | | >80 year olds | |
| --- | --- | --- | --- | --- | --- | --- |
|  | MMSE | 15WT | MMSE | 15WT | MMSE | 15WT |
| ApoE ε4 isoform | 962 | 941 | 622 | 589 | 292 | 275 |
| Total cholesterol | 494 | 475 | 432 | 401 | 177 | 160 |
| LDL cholesterol | 491 | 472 | 430 | 399 | 176 | 159 |
| HDL cholesterol | 494 | 475 | 431 | 400 | 177 | 160 |
| Homocysteine | 495 | 476 | 435 | 405 | 179 | 162 |
| Hypertension | 543 | 518 | 501 | 465 | 224 | 198 |
| DM | 1290 | 1165 | 793 | 683 | 438 | 345 |
| MI | 1287 | 1163 | 782 | 679 | 429 | 338 |
| Stroke | 1290 | 1165 | 793 | 683 | 438 | 345 |
| Depressive symptoms | 1289 | 1165 | 789 | 679 | 436 | 344 |
| CRP | 814 | 809 | 445 | 443 | 212 | 210 |
| IL-6 | 814 | 809 | 446 | 444 | 212 | 210 |
| ACT | 813 | 808 | 445 | 443 | 212 | 210 |
| Alcohol | 1166 | 1164 | 681 | 677 | 352 | 344 |
| Smoking | 1166 | 1164 | 684 | 681 | 353 | 345 |
| Physical activity | 1256 | 1142 | 747 | 656 | 407 | 328 |

*15WT* 15 Words Test (immediate recall and delayed recall had the same sample size), *ACT* a1-antichymotrypsin, *ApoE* apolipoprotein E, *CRP* C-reactive protein, *DM* Diabetes Mellitus, *HDL* High-Density Lipoprotein; *IL-6* interleukin-6, *LDL* Low-Density Lipoprotein, *MI* Myocardial infarction, *MMSE* Mini-Mental State Examination.

**Table S3. The association of hypertension with cognitive decline per age group**

| Hypertension variable | MMSE | | | 15WT immediate recall | | |
| --- | --- | --- | --- | --- | --- | --- |
|  | ≤70 | >70-80 | >80 | ≤70 | >70-80 | >80 |
| Use of antihypertensive medication | -3.99* | -1.58 | **5.02*** | **-9.99*** | **3.33*** | 0.79 |
| Blood pressure >140/90mmHg | -0.03 | -2.77 | **5.91** | -2.26 | -1.63 | 4.35 |

Shown are B’s (multiplied by 100) of the associations of a risk factor with cognitive decline within each age group. They show the extra cognitive decline (next to the overall cognitive decline as visualized in Figure 1) per age group in the presence of a risk factor. A negative B indicates that a unit increase in the risk factor is associated with more cognitive decline. Bold B’s indicate a significant (p<0.05) association with cognitive decline in that age group. *Association of risk factor with MMSE or 15WT decline is significantly different between these two age groups. B’s are determined by linear mixed models in combination with splines and adjusted for sex and education. *15WT* 15 Words Test, *MMSE* Mini-Mental State Examination.

**Table S4. Baseline characteristics of subjects still in the sample during follow-up**

| Characteristic | LASA cycle 1992-1993 | LASA cycle 1995-1996 | LASA cycle 1998-1999 | LASA cycle 2001-2002 | LASA cycle 2005-2006 | LASA cycle 2008-2009 | LASA cycle 2011-2012 |
| --- | --- | --- | --- | --- | --- | --- | --- |
| Sample size^a^ | 2527 | 2148 | 1782 | 1482 | 1122 | 891 | 708 |
| Age, y | 70.1 | 69.3 | 68.3 | 67.1 | 65.3 | 64.3 | 63.3 |
| Female, % | 51.2 | 53.0 | 54.9 | 57.1 | 58.6 | 59.3 | 60.6 |
| Education, y | 9.1 | 9.1 | 9.2 | 9.3 | 9.4 | 9.4 | 9.5 |
| Follow-up, y (median) | 9.1 | 13.0 | 14.7 | 16.2 | 19.1 | 19.1 | 19.2 |
| MMSE, points (median) | 28 | 28 | 28 | 28 | 28 | 29 | 29 |
| 15WT immediate recall, words | 5.3 | 5.4 | 5.7 | 5.9 | 6.2 | 6.3 | 6.4 |
| 15WT delayed recall, words | 8.1 | 8.2 | 8.4 | 8.6 | 8.9 | 9.0 | 9.2 |
| APOE ε4, %^b^ | 26.3 | 26.3 | 26.7 | 26.9 | 26.4 | 26.0 | 24.4 |
| Total cholesterol, mmol/L | 5.7 | 5.7 | 5.7 | 5.7 | 5.8 | 5.9 | 5.9 |
| LDL cholesterol, mmol/L | 3.7 | 3.7 | 3.7 | 3.7 | 3.8 | 3.8 | 3.8 |
| HDL cholesterol, mmol/L | 1.3 | 1.3 | 1.4 | 1.4 | 1.4 | 1.4 | 1.4 |
| Homocysteine, mmol/L | 14.5 | 14.5 | 14.2 | 13.8 | 13.5 | 13.3 | 13.0 |
| Vitamin B12, pMol/L (median) | 266 | 266 | 267 | 266 | 266 | 268 | 269 |
| Hypertension, % | 76.7 | 76.7 | 76.5 | 75.9 | 75.2 | 73.3 | 69.6 |
| Myocardial infarction, % | 8.8 | 7.8 | 6.9 | 5.9 | 5.4 | 5.1 | 4.2 |
| Diabetes Mellitus, % | 7.0 | 5.6 | 4.6 | 3.9 | 3.3 | 3.0 | 2.1 |
| Stroke, % | 4.4 | 3.9 | 3.2 | 2.2 | 1.3 | 0.6 | 0.6 |
| CES-D total score (median) | 5 | 5 | 5 | 5 | 5 | 4 | 5 |
| IL-6, pg/ml (median) | 1.4 | 1.4 | 1.3 | 1.3 | 1.3 | 1.2 | 1.2 |
| CRP, ug/ml (median) | 2.2 | 2.1 | 2.0 | 1.9 | 1.8 | 1.6 | 1.6 |
| ACT, % of NHP | 173.6 | 171.3 | 170.0 | 169.2 | 166.0 | 164.4 | 164.3 |
| Alcohol consumption, % None  Minimal^c^ Moderate^d^ | 20.1 20.6 59.4 | 19.2 20.5 60.3 | 18.1 20.0 61.9 | 17.8 20.0 62.2 | 16.9 19.7 63.4 | 15.5 20.0 64.5 | 15.2 19.2 65.6 |
| Smokers, % | 24.6 | 25.6 | 24.2 | 23.5 | 23.0 | 22.1 | 21.3 |
| Total physical activity, min per day | 169.2 | 174.6 | 179.8 | 183.4 | 190.4 | 191.4 | 196.8 |

Shown are the baseline characteristics of the subjects that are still in the sample during follow-up per Longitudinal Aging Study Amsterdam (LASA) cycle. ^a^Sample size varies per characteristic. ^b^Percentage of subjects with an apolipoprotein E ε4 isoform as proxy for an APOE ε4 allele. ^c^Women:1 drink/day, men: 1-2 drinks/day. ^d^Women: >1 drink/day, men: >2 drinks/day. Values are means unless stated otherwise. *15WT* 15 Words Test, *ACT* a1-antichymotrypsin, *APOE* apolipoprotein E, *CES-D* Center for Epidemiologic Studies Depression scale, *CRP* C-reactive protein, *HDL* High-Density Lipoprotein, *IL-6* interleukin-6, *LDL* Low-Density Lipoprotein, *MMSE* Mini-Mental State Examination, *NHP* normal human plasma.

**Table S5. The association of risk factors with MMSE in three age groups**

| Risk factor | ≤70 year olds | | | >70-80 year olds | | | >80 year olds | | |
| --- | --- | --- | --- | --- | --- | --- | --- | --- | --- |
|  | B | SE | *P-value* | B | SE | *P-value* | B | SE | *P-value* |
| APOE ε4 | 0.72 | 2.05 | *0.72* | **-4.24** | **1.81** | ***0.02*** | **-10.17** | **2.54** | ***<0.01*** |
| Total cholesterol | -3.37 | 2.43 | *0.17* | -0.10 | 1.07 | *0.93* | 1.26 | 1.13 | *0.26* |
| LDL cholesterol | **-5.40** | **2.72** | ***0.05*** | 0.66 | 1.16 | *0.57* | 1.83 | 1.24 | *0.14* |
| HDL cholesterol | 7.35 | 5.39 | *0.17* | -2.17 | 2.56 | *0.40* | -1.07 | 2.96 | *0.72* |
| Homocysteine | 0.39 | 0.44 | *0.37* | -0.40 | 0.21 | *0.05* | **-0.92** | **0.24** | ***<0.01*** |
| Hypertension | 0.04 | 5.12 | *0.99* | -2.14 | 2.37 | *0.37* | **6.52** | **2.65** | ***0.01*** |
| DM | -0.95 | 4.11 | *0.82* | -0.46 | 3.43 | *0.89* | -9.32 | 4.88 | *0.06* |
| MI | 2.42 | 3.50 | *0.49* | -1.59 | 2.81 | *0.57* | 2.23 | 3.61 | *0.54* |
| Stroke | 1.25 | 6.80 | *0.86* | -9.16 | 4.91 | *0.06* | 9.16 | 5.67 | *0.11* |
| Depressive symptoms | -0.07 | 0.11 | *0.50* | 0.02 | 0.10 | *0.84* | -0.06 | 0.13 | *0.67* |
| CRP | -0.18 | 0.17 | *0.29* | 0.18 | 0.13 | *0.18* | 0.19 | 0.15 | *0.21* |
| IL-6 | 0.02 | 0.40 | *0.97* | -0.10 | 0.31 | *0.76* | **1.31** | **0.49** | ***<0.01*** |
| ACT | 0.00 | 0.02 | *0.99* | 0.01 | 0.02 | *0.55* | **0.06** | **0.02** | ***<0.01*** |
| Alcohol^a^: minimal^b^ | 2.82 | 2.47 | *0.25* | -0.26 | 2.20 | *0.91* | **7.93** | **2.71** | ***<0.01*** |
| Alcohol^a^: moderate^c^ | 0.78 | 1.86 | *0.68* | -0.95 | 1.71 | *0.58* | 3.48 | 2.11 | *0.10* |
| Smoking | -0.30 | 1.66 | *0.86* | -1.05 | 1.78 | *0.56* | -5.15 | 2.73 | *0.06* |
| Physical activity | 0.00 | 0.01 | *0.94* | 0.00 | 0.01 | *0.60* | **0.02** | **0.01** | ***<0.01*** |

Shown are B’s, standard errors (SE), both multiplied by 100, and p-values of the associations of a risk factor with cognitive decline within each age group. They show the extra cognitive decline (next to the overall cognitive decline as visualized in Figure 1) per age group in the presence of a risk factor. A negative B indicates that a unit increase in the risk factor is associated with more cognitive decline. Analyses were performed using a linear mixed model including splines with sex and education as covariates. ^a^No alcohol use is reference group. ^b^Women:1 drink/day, men: 1-2 drinks/day. ^c^Women: >1 drink/day, men: >2 drinks/day. *ACT* a1-antichymotrypsin, *APOE* apolipoprotein E, *CRP* C-reactive protein, *DM* Diabetes Mellitus, *HDL* High-Density Lipoprotein, *IL-6* interleukin-6, *LDL* Low-Density Lipoprotein, *MI* Myocardial infarction.

**Table S6. The association of risk factors with 15WT immediate recall in three age groups**

| Risk factor | ≤70 year olds | | | >70-80 year olds | | | >80 year olds | | |
| --- | --- | --- | --- | --- | --- | --- | --- | --- | --- |
|  | B | SE | *P-value* | B | SE | *P-value* | B | SE | *P-value* |
| APOE ε4 | -0.65 | 2.09 | *0.76* | **-3.85** | **1.79** | ***0.03*** | **-5.32** | **2.71** | ***0.05*** |
| Total cholesterol | -1.08 | 2.14 | *0.62* | -1.09 | 0.96 | *0.26* | 1.53 | 1.09 | *0.16* |
| LDL cholesterol | -0.18 | 2.40 | *0.94* | -1.92 | 1.04 | *0.07* | 1.96 | 1.17 | *0.09* |
| HDL cholesterol | -1.84 | 4.76 | *0.70* | 2.64 | 2.31 | *0.25* | 0.50 | 2.82 | *0.86* |
| Homocysteine | -0.39 | 0.39 | *0.32* | -0.21 | 0.19 | *0.27* | 0.13 | 0.24 | *0.47* |
| Hypertension | -5.65 | 4.43 | *0.21* | -0.22 | 2.09 | *0.92* | **5.06** | **2.49** | ***0.04*** |
| DM | -0.11 | 4.59 | *0.98* | -3.49 | 3.66 | *0.34* | -0.29 | 5.67 | *0.96* |
| MI | 0.41 | 3.79 | *0.91* | 1.93 | 2.94 | *0.51* | 5.64 | 3.86 | *0.14* |
| Stroke | 2.60 | 7.15 | *0.72* | -0.34 | 5.32 | *0.95* | 6.70 | 6.81 | *0.32* |
| Depressive symptoms | -0.21 | 0.12 | *0.08* | 0.00 | 0.11 | *0.99* | 0.07 | 0.14 | *0.63* |
| CRP | -0.21 | 0.18 | *0.23* | 0.03 | 0.14 | *0.81* | 0.19 | 0.16 | *0.22* |
| IL-6 | 0.27 | 0.41 | *0.52* | -0.11 | 0.33 | *0.73* | 0.64 | 0.54 | *0.23* |
| ACT | -0.01 | 0.02 | *0.58* | -0.01 | 0.02 | *0.41* | 0.03 | 0.02 | *0.20* |
| Alcohol^a^: minimal^b^ | 3.19 | 2.90 | *0.27* | -3.09 | 2.36 | *0.19* | -0.31 | 2.93 | *0.92* |
| Alcohol^a^: moderate^c^ | 2.85 | 2.34 | *0.22* | -3.51 | 1.90 | *0.06* | 1.42 | 2.37 | *0.55* |
| Smoking | **-5.84** | **1.72** | ***<0.01*** | 0.53 | 1.81 | *0.77* | -1.13 | 3.00 | *0.70* |
| Physical activity | -0.01 | 0.01 | *0.31* | 0.01 | 0.01 | *0.17* | 0.00 | 0.01 | *0.72* |

Shown are B’s, standard errors (SE), both multiplied by 100, and p-values of the associations of a risk factor with cognitive decline within each age group. They show the extra cognitive decline (next to the overall cognitive decline as visualized in Figure 1) per age group in the presence of a risk factor. A negative B indicates that a unit increase in the risk factor is associated with more cognitive decline. Analyses were performed using a linear mixed model including splines with sex and education as covariates. ^a^No alcohol use is reference group. ^b^Women:1 drink/day, men: 1-2 drinks/day. ^c^Women: >1 drink/day, men: >2 drinks/day. *ACT* a1-antichymotrypsin, *APOE* apolipoprotein E, *CRP* C-reactive protein, *DM* Diabetes Mellitus, *HDL* High-Density Lipoprotein, *IL-6* interleukin-6, *LDL* Low-Density Lipoprotein, *MI* Myocardial infarction.

**Table S7. The association of risk factors with 15WT delayed recall in three age groups**

| Risk factor | ≤70 year olds | | | >70-80 year olds | | | >80 year olds | | |
| --- | --- | --- | --- | --- | --- | --- | --- | --- | --- |
|  | B | SE | *P-value* | B | SE | *P-value* | B | SE | *P-value* |
| APOE ε4 | 0.18 | 2.32 | *0.94* | **-4.15** | **2.00** | ***0.04*** | **-8.52** | **3.07** | ***<0.01*** |
| Total cholesterol | 0.97 | 2.40 | *0.68* | -1.53 | 1.08 | *0.16* | 1.27 | 1.25 | *0.31* |
| LDL cholesterol | 0.68 | 2.69 | *0.80* | **-2.62** | **1.17** | ***0.03*** | 1.41 | 1.33 | *0.29* |
| HDL cholesterol | 6.21 | 5.32 | *0.24* | 1.45 | 2.60 | *0.58* | 2.97 | 3.26 | *0.36* |
| Homocysteine | -0.76 | 0.43 | *0.08* | -0.08 | 0.21 | *0.79* | 0.13 | 0.27 | *0.64* |
| Hypertension | -4.04 | 4.93 | *0.41* | 3.54 | 2.34 | *0.13* | 0.43 | 2.86 | *0.88* |
| DM | -1.68 | 5.12 | *0.74* | -5.44 | 4.09 | *0.18* | 4.90 | 6.47 | *0.45* |
| MI | 4.90 | 4.23 | *0.25* | 0.62 | 3.31 | *0.85* | **10.14** | **4.34** | ***0.02*** |
| Stroke | 0.79 | 8.01 | *0.92* | -1.58 | 6.01 | *0.79* | -4.30 | 7.64 | *0.58* |
| Depressive symptoms | -0.11 | 0.13 | *0.41* | -0.23 | 0.12 | *0.05* | 0.21 | 0.16 | *0.20* |
| CRP | -0.19 | 0.19 | *0.33* | -0.11 | 0.15 | *0.47* | 0.16 | 0.89 | *0.38* |
| IL-6 | 0.31 | 0.45 | *0.50* | -0.03 | 0.37 | *0.93* | 0.88 | 0.60 | *0.14* |
| ACT | -0.01 | 0.02 | *0.71* | -0.04 | 0.02 | *0.06* | 0.03 | 0.03 | *0.21* |
| Alcohol^a^: minimal^b^ | 5.91 | 3.23 | *0.07* | -3.03 | 2.65 | *0.25* | 0.25 | 3.36 | *0.94* |
| Alcohol^a^: moderate^c^ | 3.19 | 2.61 | *0.22* | -0.47 | 2.13 | *0.83* | -0.69 | 2.71 | *0.80* |
| Smoking | **-4.52** | **1.92** | ***0.02*** | -2.88 | 2.04 | *0.16* | -0.61 | 3.37 | *0.86* |
| Physical activity | 0.01 | 0.01 | *0.45* | 0.01 | 0.01 | *0.32* | 0.00 | 0.01 | *0.75* |

Shown are B’s, standard errors (SE), both multiplied by 100, and p-values of the associations of a risk factor with cognitive decline within each age group. They show the extra cognitive decline (next to the overall cognitive decline as visualized in Figure 1) per age group in the presence of a risk factor. A negative B indicates that a unit increase in the risk factor is associated with more cognitive decline. Analyses were performed using a linear mixed model including splines with sex and education as covariates. ^a^No alcohol use is reference group. ^b^Women:1 drink/day, men: 1-2 drinks/day. ^c^Women: >1 drink/day, men: >2 drinks/day. *ACT* a1-antichymotrypsin, *APOE* apolipoprotein E, *CRP* C-reactive protein, *DM* Diabetes Mellitus, *HDL* High-Density Lipoprotein, *IL-6* interleukin-6, *LDL* Low-Density Lipoprotein, *MI* Myocardial infarction.
