# Supplementary material for: Age dependency of risk factors for cognitive decline
Source: BMC Geriatr. 2018 Aug 20;18:187. doi: 10.1186/s12877-018-0876-2 (PMC6102935; doi:10.1186/s12877-018-0876-2)
Supplement: Supplementary file 2 — Additional explanation about the spline regression analyses. (DOCX 375 kb) [file 12877_2018_876_MOESM2_ESM.docx]

**Additional file 2: Spline regression analyses**

Spline regression analyses – method

Splines convert the continuous age variable into pieces by using piecewise functions. These piecewise functions join smoothly at certain points, which are called knots. Spline regression models are preferred over models with categorized data. Categorical models assume the association between age and outcome to be constant within categories, whereas splines use all the data points and estimate the association in the total age range.

We tested both linear as cubic splines using one or two knots. Linear splines estimate a linear association between the knots, whereas cubic splines assess a cubic (non-linear) function.

Linear mixed models including the splines were used to determine the association of the risk factors with the three cognitive outcome measures in the total sample. To assess the age dependency of the risk factors, we added an interaction of the risk factors with the splines to the model. To keep this model manageable, random intercepts and fixed slopes were used. Lastly also the association of the risk factors with the cognitive outcome measures per age group were determined. All the analyses were adjusted for sex and education (in years).

Spline regression analyses – results

The final model consisted of linear splines with two knots at the ages of 70 and 80 years for all three outcome measures. As an example, we visualized the association between MMSE and age in Figure S1 (see Figure 1A in the manuscript) and in Figure S2 the association between age and MMSE for subjects with and without hypertension at baseline (see Figure 2A in the manuscript). We added the decline in MMSE per age group in both figures which helps to interpret the results (see Additional file 1 – Table A3 and Table 3 in the manuscript).


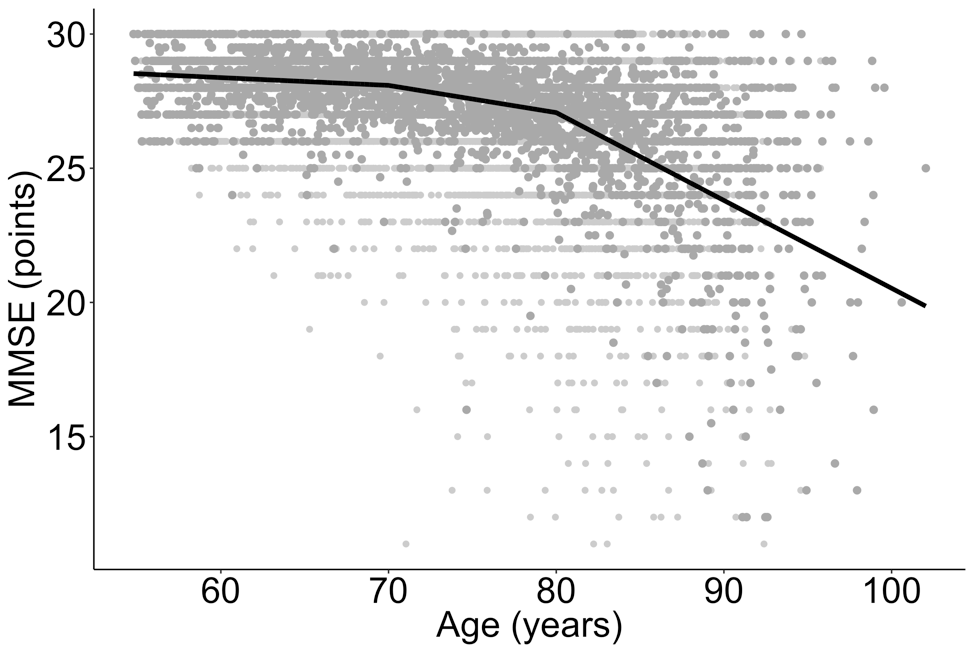


-0.18

-0.06

-0.25

**Figure S1**. Association between age and Mini-Mental State Examination (MMSE). The grey dots represent all the longitudinal data points. The lines represent the splines with the two knots (black dots) at the ages 70 and 80 years. The numbers represent the change in MMSE per year (see Table 3 in the manuscript).

-2.14

0.04


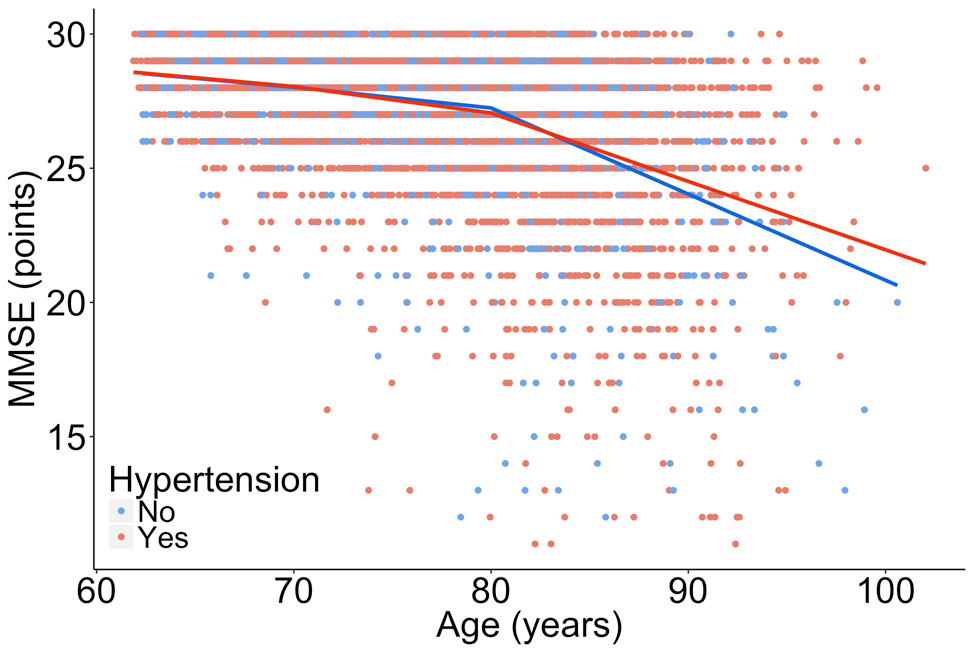


6.52

**Figure S2**. Association between age and Mini-Mental State Examination (MMSE) for subjects with and without hypertension at baseline. The numbers represent the extra decline (so in addition to the decline as visualized in Figure S1) in MMSE (multiplied by 100) per age group in the presence of hypertension (see Table 3 in the manuscript).
